# Potential of Dietary Microalgae as Feed Additive in Japanese Quail

**DOI:** 10.1155/vmi/6641503

**Published:** 2026-09-16

**Authors:** Tahreem Fatima, Mamoona Tahir, Aqsa Hameed, Faiza Shahzadi, Shabana Naz, Amna Sajjad, Maleeha Manzoor

**Affiliations:** ^1^ Department of Zoology, Faculty of Life Sciences, Government College University Faisalabad, Faisalabad, Pakistan, gcuf.edu.pk

**Keywords:** carotenoids, dietary microalgae, growth performance, immune response, oxidative stress, quails

## Abstract

Due to substantial increase of human population, demand for sustainable protein sources of livestock, poultry, and fish also increases. To meet the escalating demand for protein‐rich food, there is also need of finding suitable feed ingredients to improve the growth and health of poultry especially quails. Employing microalgae as feed may promote increased health and digestive system functions. Microalgae are considered as a sustainable alternative to the conventional feed due to their unique nutritional composition, high protein content, rich in essential fatty acids minerals, antioxidants and vitamins, and thus have ability to improve quails diet. Additionally, microalgae improve health quality by enhancing immune status, reducing oxidative stress, and enhancing antimicrobial benefits. Thus, incorporating microalgae supplementation to quail feed enhances meat and egg quality, improves growth performance, and promotes digestive system functions.

## 1. Introduction

Poultry is an important constituent as a source of protein in diet of rural people whose traditional foods are rich in carbohydrates [1]. Around the world, poultry products are a cheap source of animal protein. To maintain a consistent supply of protein for the growing human population, poultry production must be improved. This involves an establishment of strategies that might improve poultry’s productive performance. Various feed additives, including exogenous enzymes, probiotics, and phytogenic supplements, are included in nutritional methods [2]. Therefore, scientists and farmers are highly interested in the investigation of new sources of protein and energy for poultry. Almost 30% of globally produced microalgae are used as feed for animals, making microalgae biomass a highly effective feed choice for animals [3]. It is also documented that biologically active supplements, including phycocyanin, along with gamma‐linolenic and phenolic compounds in *Spirulina platensis*, are mainly responsible for antibacterial, immunostimulant, disease‐resistant, and antioxidant properties [4].

Japanese quail is one of the most significant species in the poultry industry from an economic perspective because of its unique physiological traits, which include swift growth, efficient reproduction, a brief life cycle, and early sexual maturity [5]. The Japanese quail is classified under the order Galliformes, family Phasianidae, genus *Coturnix*, and species *japonica*. Its accepted scientific name is *Coturnix japonica*, which distinguishes it from the common quail, *Coturnix coturnix*. Historical records indicate that wild Japanese quails were first documented in Japan during the eighth century. Adult females possess pale breast feathers marked with dark speckles, whereas adult males possess uniform dark rust‐red coloration on the breast [6] Japanese quails were initially domesticated as a songbird but later gained prominence as a food‐producing species. Its growing utility is due to several factors such as its economic value. Consumption of Japanese quail eggs and meat is believed to provide medicinal benefits and enhance resistance to various diseases [7]. Though meat is the primary foodstuff in Europe, egg production is significant in Southeast Asia [8, 9]. The smallest bird species raised for meat and eggs is the Japanese quail [10].

Due to its unique traits in comparison to other poultry species, *Coturnix coturnix japonica* is increasingly recognized as the best animal for experimentation [10]. The quails can produce multiple generations quickly, have high egg rates, and require little space for feeding and care [11]. Recently, Japanese quails are being used in welfare studies to measure several physiological, biochemical, behavioral, and productive parameters considered indirect indicators of the welfare of birds [12]. Additionally, the production of meat as well as eggs from *C. japonica* (Japanese quail) offers a significant amount of animal protein, and it is preferred by customers in certain nations, regarded as a cheap meal [13]. As compared to other poultry products, quail meat is highly valued for its superior nutritional profile, which includes high‐quality protein, low fat, high biological value, and less calories [14], and requires less space and feed than chicken. As a result, there is growing interest in optimizing the composition of quail meat by lowering fat content and pH while improving fatty acid profiles. The goal of quail breeders is to produce high‐quality meat in less time and at the lowest possible cost. Moreover, it is also documented as a substitute of protein source for human welfare, especially in developing countries [15].

## 2. Literature Search Strategy

A literature search was carried out to find related studies about the supplementation of quails with microalgae. Electronic databases such as PubMed, Google Scholar, and Scopus were searched with key words such as “*Chlorella*,” “*Spirulina*,” “Japanese quail,” “poultry growth performance,” and “microalgae supplementation.” The publications from the year of 2000 to 2026 were analyzed. The criteria for inclusion were peer‐reviewed original research articles and reviews in English reporting results of microalgae supplementation on biochemical or physiological parameters on poultry growth. Studies were excluded if they did not include any poultry species, or if they were not available as full text or not peer reviewed.

## 3. Nutritional Composition of Microalgae

**FIGURE 1:**
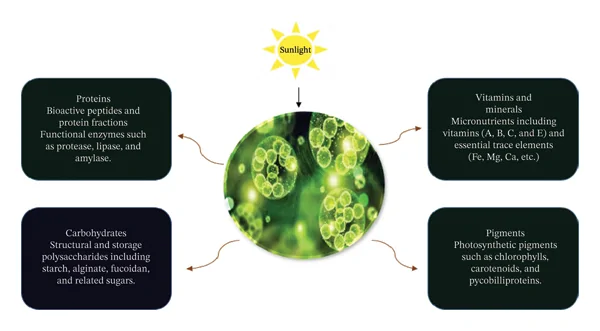
The nutritional composition of algal cells.

Microalgal biomass comprises proteins, essential amino acids (EAAs), lipids, carbohydrates, pigments, vitamins, and minerals, many of which have antioxidant or immunomodulatory functions in animals [16] (Figure 1).

Microalgae comprise diverse taxa, mainly within Chlorophyta (green microalgae such as *Chlorella*, *Scenedesmus*, and *Dunaliella*) and Cyanobacteria (*Arthrospira*/*Spirulina*). Among these, *Chlorella vulgaris* and *Chlorella sorokiniana* are the most studied for poultry feed because of their high protein and carotenoid content [17]. Microalgal nutritional composition varies with cultivation systems (open ponds vs. photobioreactors), nutrient supply, light intensity, and harvest timing. These parameters influence cellular protein and lipid and carbohydrate contents and therefore increase overall feed value. The rigid cell wall of *Chlorella* can limit digestibility; however, mechanical or enzymatic cell‐wall disruption significantly improves nutrient bioavailability in poultry diets [18]. Depending on species and growth conditions, *Chlorella* typically contains 40%–60% protein, 5%–20% lipids, and 10%–25% carbohydrates. Proteins comprise of all EAAs, while its lipids include valuable polyunsaturated fatty acids such as α‐linoleic acids. Carotenoids and phenolics reduce lipid peroxidation in tissues and meat products [19].

Polysaccharides and oligosaccharides act as prebiotics in cell walls by modulating gut microbiota and immune cell activation. *Chlorella*‐derived peptides and nucleic acids, sometimes called *Chlorella* growth factor, have been linked to enhanced cellular repair and immune responses. Studies show that *Chlorella* supplementation increases serum antioxidant enzyme activities such as catalase (CAT), superoxide dismutase (SOD), and glutathione peroxidase (GPx) and decreases malondialdehyde (MDA), demonstrating its role in mitigating oxidative stress [17].

**FIGURE 2:**
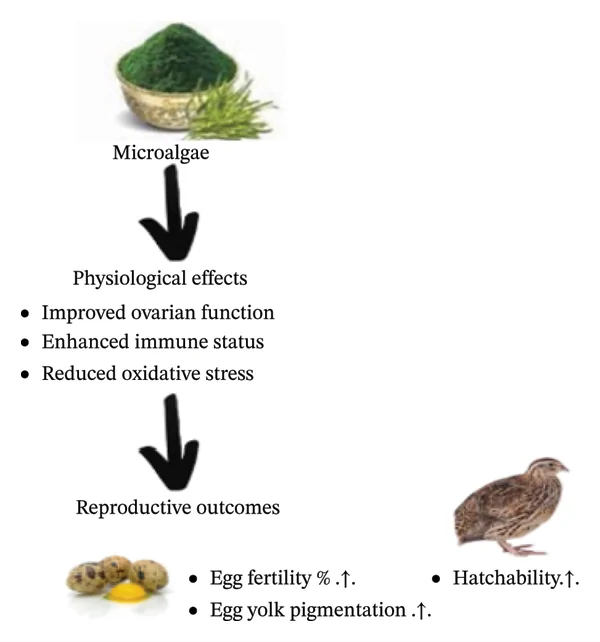
The beneficial impacts of microalgae supplementation on various poultry species, e.g., broiler, quail, and chick.

Microalgae are extensively used in poultry nutrition due to their roles as natural feed additives, providing essential nutrients and bioactive compounds. They serve as rich sources of pigments, essential fatty acids, amino acids, and antioxidants, which contribute to improved growth performance, immune modulation, and meat quality [3, 20]. In addition, the incorporation of microalgal biomass such as *Spirulina platensis*, *C. vulgaris*, and *Schizochytrium* sp. in poultry feed improves gut health and oxidative stability while reducing the need for antibiotic growth promoters [21]. Microalgae‐based diets show beneficial effects in poultry, highlighting their nutritional, immunological, and environmental advantages within sustainable livestock production systems (Figure 2).

## 4. Functional Properties of Microalgae

### 4.1. Antioxidant Potential

Microalgae are a rich source of carotenoids such as β‐carotene, astaxanthin, lutein, and lycopene. These compounds provide characteristic pigmentation to the cells and play an important role in cell protection against oxidative damage by free radicals. The potency of these antioxidants is much higher than that of vitamin E. Microalgal‐derived antioxidants exhibit significant potential as dietary supplementation to enhance poultry health [22]. Poultry is exposed to oxidative pressure caused by the formation of reactive oxygen species (ROS). To cope with this stress, they have evolved efficient defense strategies that involve the synthesis and accumulation of diverse antioxidant systems [23]. These antioxidant defenses include phenolic compounds, carotenoids, nitrogen‐containing molecules, and dimethyl sulfoxide. Microalgal species such as *Spirulina*, *Schizochytrium*, and *Isochrysis* are suitable for a wide range of biological and industrial applications due to strong antioxidant capacities [24].

Microalgae can significantly reduce oxidative stress in quails. Research indicates that adding microalgae to quail’s diet lowers MDA levels, which is a marker of cell damage [25]. It also boosts their own natural defense enzymes, such as SOD and CAT. Moreover, quails being fed on microalgae have improved quality of their meat and eggs. Quails take pigments and polyunsaturated fatty acids from microalgae and accumulate them in their eggs [26]. Therefore, microalgae‐supplemented feed is a practical way to help quails stay healthy and resilient, even in challenging environments.

### 4.2. Antimicrobial Activities

The problem of increasing microbial resistance because of large antibiotic consumption has led to serious scientific studies on alternative antimicrobial agents. Metabolites obtained from extremophile microalgae possess antimicrobial properties [27]. Various microalgal species such as *Spirulina platensis*, *Chlorella vulgaris* and *Scenedesmus* sp. contain strong antibacterial activity. Bioactive molecules like chlorellin, a compound produced by *Chlorella*, demonstrates significant inhibitory effects against a wide range of organisms [28]. Fatty acids derived from various microalgae exhibit significant antibacterial potential against several bacterial strains [29].

The effect of *Spirulina platensis* supplementation on gut microbiota has been widely studied in poultry. Emerging evidence highlights its importance in quails, particularly under heat stress conditions. Dietary inclusion of *Spirulina* at different levels has been linked with a reduction in pathogenic bacterial populations. The levels of *Escherichia coli* in the ileal content were affected, while populations of beneficial *Lactobacillus* species remained stable. This targeted antimicrobial effect suggests that *Spirulina platensis* can regulate gut microbial balance without disrupting commensal flora [30]. New experimental evidence supports the antimicrobial effectiveness of *Spirulina* against poultry pathogens at the cellular level. For instance, extracts from *Spirulina platensis* have shown inhibitory activity against antibiotic‐resistant strains of *Mycoplasma gallisepticum*, a major respiratory pathogen in poultry [31]. The findings highlight the role of microalgae as a naturally occurring antimicrobial substance with a different mode of action compared to conventional antibiotics.

### 4.3. Anti‐Inflammatory Activity

Anti‐inflammatory properties of microalgae have been identified in saccharides, peptides, enzymes, and polyunsaturated fatty acids found within microalgae [32]. High concentrations of omega‐3 polyunsaturated fatty acids, specifically docosahexaenoic acid (DHA) and eicosapentaenoic acid (EPA), are the primary cause of the anti‐inflammatory impact of microalgae in poultry. It also contains specialized pigments like astaxanthin and phycocyanin. These compounds modulate inflammatory responses by inhibiting the NF‐κB signaling pathway. This action reduces the production of proinflammatory cytokines and tumor necrosis factor [33]. Additionally, microalgae support improved gut health by modulating the intestinal microbiota and enhancing the stability of the intestinal barrier. As a result, systemic inflammation is reduced and nutrient absorption is improved in birds that are being reared under challenging environmental conditions [34].

## 5. Effects on Growth Performance

The microalgae supplementation of the diet has a significant positive impact on the growth performance of Japanese quails, increasing the body weight and weight gain relative to controls [35]. *Spirulina* addition demonstrates a high linear growth in weight gain, lower intake of feed, and lower feed conversion ratio (FCR), which is desirable feed efficiency [36]. The optimal level of FCR is achieved at lower levels (0.5 g/kg), whereas higher levels (1 g/kg) increase early growth performance [37]. Although there is a certain difference between growth phases, treatment has a statistically significant effect on the body weight gain, feed intake, and FCR [37]. Nevertheless, the inclusion of *Spirulina* and propolis can decrease the amount of feed and weight gain, yet FCR remains positive [30]. In general, microalgae, especially *Spirulina*, can be used as efficient feed additives to enhance growth efficiency without the impact on mortality [30].

Recent findings have highlighted the promising role of microalgal supplementation in improving growth performance parameters in quails. It has been shown to enhance live body weight and overall body weight gain, with the most notable improvements observed at moderate inclusion levels (e.g., 0.5 g/kg of feed). Although the influence on feed intake was generally nonsignificant across different growth stages, the FCR demonstrated significant improvement, suggesting better feed utilization efficiency when microalgae were incorporated into the diet (Table 1).

**TABLE 1:** Impact of different microalgal species on quails.

Microalgae species	Dose (% or g/kg)	Quail age	Growth performance outcomes	Other effects	References
*Dunaliella salina*	0.25, 0.5, and 1 g/kg	7‐day‐old Japanese quail chicks (up to 5 weeks)	Improved body weight and live body weight enhanced FCR at 0.5 and 1 g/kg, and no change in feed intake	Improved kidney response at 0.5 g/kg and liver indicators at 1 g/kg, a stronger complement‐3 immunological response, and a better lipid profile (↓ TC, LDL, and TG; ↑ HDL) at 0.5 and 1 g/kg.	[25]

*Spirulina platensis* powder	1.5%, 3.0%, 4.5%, 6.0%	Japanese quail chicks (10‐day old) up to 38 days	4.5% group outperformed the control in terms of FCR, growth, and live weight.	3% showed the best immunological response and antioxidant status; highest *Lactobacillus sp.*in 6% and lowest *E. coli/Salmonella* in 1.5% and 4%; and overall digestive enzymes increased (highest trypsin and amylase in 3% and highest lipase in 4.5%).	[38]

*Dunaliella salina* + *Arthrospira platensis* mix (DsAp)	DsAp0.5: 0.25 kg/g each; DsAp1: 0.5 kg/g each; DsAp2: 1 g/kg each	7‐day quails, up to 5 weeks	The highest live weight (∼191 g) and body weight gain (∼5.69 g/day) were attained by DsAp1 (0.5 + 0.5 g/kg); DsAp0.5 showed decrease in feed intake (19.63 g) but it had the best FCR.	DsAp0.5 showed best economic returns. Good lipid profile (DsAp0.5:TC & LDL↓, DsAp1: HDL↓ DsAp2: TG↓) DsAp2 shows the beneficial improvement on the liver and kidneys; no discernible changes in the immunological markers C3 and lysozyme	[35]

*Spirulina platensis*	0.5 and 1 g/kg	2‐day‐old quail chicks, 5‐week trial	Weight gain and feed intake significantly increased at 0.5 g/kg; both dosages enhanced growth compared to control.	Carcass yield (hot and cold) rose, liver weight also increased	[37]

Microalgae that have high beta‐carotene content promote reproduction in quails. Increase in growth of quail chicks could be due to changes in the gut‐friendly microbiota and immunology [39]. Microalgae enhance the dimension of ileum and villi and duodenum sections in the quails, and they improved liver’s metabolism of carotenoids [40]. Microalgae are utilized as an important supplement to improve health performance and meat quality in quails because they offer substantial nutritional benefits and enough powerful natural antioxidants, such beta‐carotene and zeaxanthin [41]. Microalgae contain proteins, carbohydrates, and fats that promote growth and metabolism, which results in improved weight of body [42].

Supplementation of microalgae in quails shows improvements in quail growth performance but it depends on the level of supplementation. In many cases, moderate inclusion rates yielded more consistent and favorable results than higher inclusion rates. For example, body weight and feed conversion ratio (FCR) were boosted by the supplementation of *D. salina* at a rate of 0.5–1 g/kg while that of *Spirulina platensis* was at higher concentration (3%–4.5%). These differences are probably related to variations in the nutritional profile and bioactive compounds of each microalgal species. *D. salina* has been reported to have a high content of β‐carotene and antioxidant pigments, while *Spirulina* contains high levels of proteins, phycocyanin, and EAA and different types of metabolites that can affect metabolism in different ways.

**FIGURE 3:**
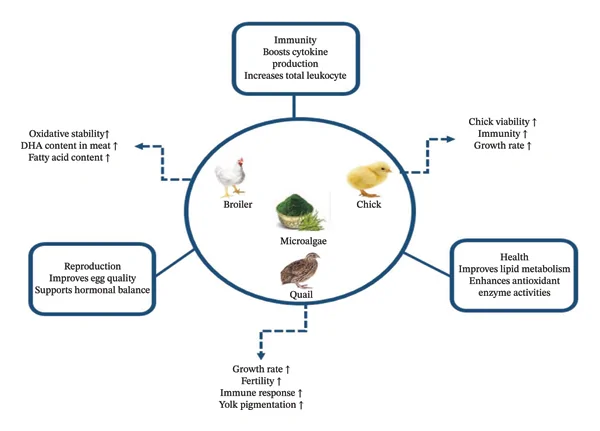
The functional contributions of microalgae in quail diet, their role as a protein source, and improvement in nutrient digestion.

Algae generate immunostimulatory exopolysaccharides with strong complement activity, which generally benefits animals. This implies that quails fed microalgae have better health outcomes due to their higher complement action [43]. The carcass characteristic is unaffected by microalgae. Japanese quails aged 15–35 days that were fed up to 8% of microalgae in feed of quails showed no effects on carcass composition or quality of meat [44]. When green microscopic algae were added to the diet of quail, the blood TP level increased, and it was suggested that feeding green microalgae might improve liver function. Brown algae include fucoidan, and related chemicals are thought to be prebiotics [45, 46]. Supplementing quails with microalgae improved their reproductive characteristics (Figure 3).

The ability of phycocyanin and fucoidan to absorb free radicals probably helps protect the kidneys by avoiding harm to the glomerular and tubular cells. This implies that these compounds might be advantageous for quail health [47].

Supplementation of microalgae results in increased serum total protein and albumin levels as the dietary concentration of phycocyanin increased in diet [48]. Nutritional microalgal supplementation in quails can improve the immune function, intestinal permeability, antioxidant capability, and cecal microbiota balance. Microalgae supplementation at a level of 0.5% enhanced the antioxidant potential detected by lowest MDA in Japanese laying quails [49]. The presence of a variety of antioxidant chemicals, such as beta‐carotene, PC, FUC, and others, may be linked to the quail’s microalgae’s antioxidant action.

## 6. Effects on Immunity and Health

Microalgae sources are rich in bioactive compounds including phycocyanin, carotenoids, polyunsaturated fatty acids, vitamins, and antioxidant pigments that when added to the diet of Japanese quail *C. japonica* can have a beneficial effect on their immunity and health [38, 50]. These functional nutrient factors not only help maintain normal physiological functions but also enhance the birds’ resistance to the environmental and metabolic stress factors. A recent study that evaluated the effect of *Spirulina* supplementation in Japanese quails showed that the antioxidant capacity, serum immunoglobulin concentration, lysozyme activity, and complement activity of quails are increased while the relative weights of bursa of Fabricius, thymus, and spleen are also improved [36]. In addition, phycocyanin obtained from *Spirulina* showed a positive effect on blood parameters, intestinal morphology, antioxidant capacity, and expression of immunologic‐related genes, indicating a beneficial effect of phycocyanin in quail and its immune response [51, 52]. The results indicate that microalgae can be used as a nutritional supplement to enhance physiological resilience and enhance resistance to different stress levels in Japanese quail [53]. Moreover, poultry studies have shown that microalgae can contribute to maintaining gut integrity and stability, reducing oxidative stress in the gut, and enhancing nutrient utilization efficiency associated with immunity competence [54, 55]. In line with this, *C. vulgaris* was demonstrated to upregulate genes associated with intestinal barrier integrity such as occludin and increase immune cell functions indicating its potential role in imparting similar immune benefits in Japanese quail [56]. Therefore, feeding microalgae to Japanese quail as supplemental feed would be a nutritional benefit to improve the health, immune status, and adaptability of quail to the challenging production environment [25, 36].

Microalgae‐based feed boosts immunity to reduce inflammation, preserve normal flora, and remove harmful microbes [57]. Immunity in Japanese quail can be initiated [38] and boosted by some supplements of microalgae, such as β‐carotene [57]. Microalgal components can protect against inflammatory infections through the enhancement of immune functions. In quails treated with microalgae, white blood cell counts and macrophage phagocytic activity are significantly enhanced, which boosts the immune system. Supplements of microalgae, including β‐carotene and vitamin B12, which seem to have immunomodulatory effects on quail immunological responses, have also been linked to improvements in quail immune systems and behavioral patterns. Microalgae directly impact the growth of quails and the stimulation of lymphatic tissues (Table 2). According to reports, adding *Spirulina platensis* microalgae to quail’s diet can boost their blood levels of IgM, IgG, phagocytic activity, and phagocytic index, all of which can lead to an immunological positive response [61].

**TABLE 2:** Impact of microalgae on immune system of quails.

Microalgae	Species	No. of species	Duration	Humidity and temperature	Performance of immune system	References
*Arthrospira platensis*	Japanese quail	480	5 weeks	50 ± 5% and 28 ± 2°C	Increased immunity, antibody production, and disease resistance	[58]

*Spirulina platensis*	Japanese quail	250	5 weeks	60%–70% and 20°C	Increased antibody production against Newcastle disease but did not impact avian influenza immunity	[52]

*Spirulina platensis*	Japanese laying quail	250	12 weeks	50%–60% and 18°C–20°C	Enhanced the antibody production and higher cutaneous basophil hypersensitivity	[59]

*Arthrospira platensis* + *Dunaliella salina*	Quail chicks	240	5 weeks	65% and 34 ± 1°C	Increased the immunity and improved liver and kidney functions	[35]

*Spirulina platensis*	Japanese quails Fertilized eggs	240	3 weeks	55%–70% and 37.5°C–37.8°C	Improved antioxidant and immunity‐related gene expression in quail chicks	[60]

In addition to improving nonspecific immune response, microalgae can also increase cell‐mediated immunity and had an immunomodulatory impact by making animals more resistant to illnesses. The use of microalgae in conjunction with vaccination improved the innate immune response that followed immunizations. These notable stimulatory effects are due to the presence of high molecular weight polysaccharides (Immulina) and Braun‐type lipoproteins [62]. Gut microbiota is favored by the acidic polysaccharides (82% DW) found in cell walls of many microalgae. *D. salina* is another intriguing microalga that is said to produce extracellular polysaccharides with antiviral, anticancer, and immunostimulant qualities. Consequently, microalgae help quails indirectly by fostering the gut microbiota, which in turn improves the animal’s health, in addition to directly supplying nutrients that boost bird performance and health [63].

A microalgae polysaccharide extract has been demonstrated to boost white blood cells in an irradiated hematopoietic system. When microalgae were given orally to healthy quails, the isolated natural killer cells (NK cells) activated with interleukin (IL)‐12/18 showed increased phagocytic activity and interferon gamma (IFN‐γ) production. Besides this, microalgae also increased the level of IL‐12 from peripheral blood mononuclear cells mediated by toll‐like receptor (TLR)–2 and 4. This suggests that microalgae first stimulate monocytes and macrophages to create cytokines that stimulate NK cells [64].

## 7. Effects on Meat Quality

Quail meat is reported to be a nutritionally superior dietary protein source having a favorable protein to fat ratio, low cholesterol level, and high protein digestibility. Quail’s feed supplemented with some level of microalgae, particularly *Spirulina platensis* and *C. vulgaris*, at measurable amounts can have a regulatory impact on the yield parameters of the carcass [65, 66]. The use of microalgal biomass has been demonstrated to improve dressing percentage and final live body weight, resulting in higher amounts of valuable breast and leg muscle in quails [67]. The crude protein percentage for microalgae varies between 50% and 70% of dry matter, and it is an exogenous protein source that directly stimulates the deposition of protein in skeletal muscle [26]. Concurrently, the use of microalgae in treatment helps to reduce the accumulation of abdominal fat and the content of intramuscular lipids, which can be explained by the regulation of de novo lipogenesis in the liver and inhibition of the synthesis of very‐low‐density lipoprotein (VLDL) [54, 68]. The presence of microalgae in quail’s feed generates a well‐balanced source of EAAs, particularly lysine and methionine, which are usually lacking in the traditional cereal‐based feed, and thus raises the muscle protein synthesis (MPS) rate [26, 68].

Microalgae can significantly affect the color indices of meat in a dose‐dependent manner, with a dose‐dependent increase in yellowness (b) and redness (a) values [51]. Regarding the meat texture, the use of microalgae always showed the best texture of quail meat (as it was evaluated by the decrease in shear force values—obtained by instrumental analysis) and the best tenderness score. This structural improvement is explained by the simultaneous increase of water holding capacity (WHC) and decrease in thermally‐induced cooking loss. Microalgae species are naturally abundant in high amounts of long‐chain poly unsaturated fatty acids (LC‐PUFAs) including gamma‐linolenic acid (GLA), EPA, and DHA [69]. The targeted alteration in the composition of phospholipids in muscle tissue through incorporation of these species leads to a proportionally decreased saturated fatty acid (SFA) fraction and a decreased ratio of n‐6 fatty acids to n‐3 fatty acids (n‐6/n‐3) [68]. *Spirulina* and *Chlorella* possess high intracellular concentrations of α‐tocopherol, ascorbic acid, C‐phycocyanin, and polyphenolic compounds that are deposited in the muscle tissue which are also systemically absorbed to scavenge ROS and to inhibit propagation reactions in lipid radicals [54, 67].

In Iran, the poultry business is the largest business community and generates the most revenue of any agricultural sector. Since the 17th century, Japanese quails have been utilized for meat [70]. The meat of quail is nutritious due to its high protein content, low cholesterol, beneficial fatty acid profile, and mineral content [71]. Furthermore, it is considered as an alternate protein used for human development [15]. EAAs are also present in breasts and the meat of the leg. A variety of minerals, such as calcium, phosphorus, sodium, magnesium, and zinc, which are beneficial for human health, are also found in quail meat [72].

Due to its high protein content and lower fat content as compared to corresponding red meat like mutton and white meat like broiler chicken, quail meat is preferred among the meat consumers [73]. The meat of Japanese quail is obtained after the quail reaches 42 days of age and the total body weight is 150 to 300 g [74]. Despite having less fat than meat from other poultry, quail meat contains unsaturated fatty acids in very large amount [75]. These fatty acids have antithrombotic and atherosclerotic properties that are good for human health [76]. Eating two quails a day gives the body 27–28 g of protein, which contains eleven grams of important amino acids. This is equivalent to consuming 125–130 g of pure meat. They also supply phenylalanine, tyrosine, and valine needed daily by humans according to their age and physiological and physical activity state. Thus, quail meat can also be considered an important protein source food, especially EAAs. Added to this is the fact that quail meat is lean with little fat content; hence, it is an excellent and relatively cheap source of animal protein. Little research has been done to examine how quail performance is affected by breeds, strains, and production types, despite the fact that it is well known that quails have a variety of plumage colors. Furthermore, little is known about the meat profiles of the many quail strains [13].

**FIGURE 4:**
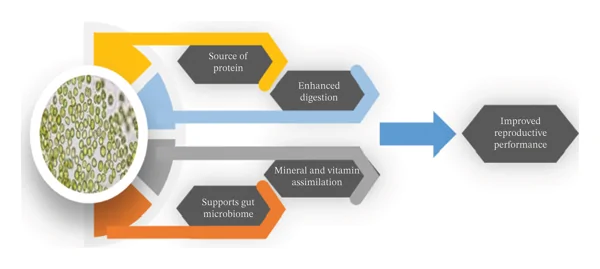
Schematic representation showing the role of *Spirulina* sp. in improving quail productivity.

Phycocyanin, a member of the phycobiliprotein family, is found in the cyanobacteria *A. platensis,* also referred to as *Spirulina*, which is grown all over the world. Brown macroalgae also comprise bioactive polymers that are sulfated and rich in fucose which can easily be extracted from their cell walls. These are known as fucoidans [77]. Indicators of the increased antioxidant capacity are increased serum total SOD with decrease in MDA content [78]. *Spirulina* sp. improves the pigmentation in egg yolk of quails (Figure 4).

Additionally, previous studies explored that the reproduction rate of laying poultry can be enhanced by algae and their derivatives [79]. It is crucial to investigate the interaction of *Arthrospira platensis* and *D. salina* in the quail’s diet, provided with their nutritional supplements. Active compounds and main components of the diet, such as the antioxidant beta‐carotene, polyphenol, and chlorophyll, are primarily responsible for *D. salina’s* biological features [80]. A recent study proved that supplementing with *A. platensis* and *D. salina* increases the physiological performance of quails (Table 3). Microalgae supplements used in the feed of Japanese quail improve meat quality and carcass quality features [35].

**TABLE 3:** Impact of microalgal species on the meat quality of quails.

Microalgae	Species	No. of species/groups	Weeks	Dose (g/kg or %)	Meat quality	References
*Spirulina platensis*	Japanese quails (*Coturnix coturnix japonica*)	250/5 replicates	5	SP1: 2.5% SP2: 5% SP3: 7.5%	Improved the meat quality and positive effect on growth performance: at 7.5%, increased feed intake and FCR, at 2.5%, total body weight increased,	[81]

*Arthrospira platensis*	Japanese quails	60 (30 males and 30 females)/5 replicates	∼3	1% 2% 4% 8%	Enhanced the meat quality and growth performance and carcass composition At 8%, adverse effects on feed intake FCR and body weight gain but shows best effect at 4%	[82]

*Spirulina platensis* (phycocyanin) Brown seaweed (fucoidan)	Japanese quail	250/5 replicates	5	PC20: 20 mg/L PC PC40: 40 mg/L PC FUC20: 20 mg/L FUC FUC40: 40 mg/L FUC	Enhanced growth performance, egg, and meat quality At PC40, decreased FCR	[83]

*Spirulina platensis*	Japanese quail (brown and white varieties)	300/5 replicates	∼ 6	T1: 2.5 g/kg T2: 5.0 g/kg T3: 7.5 g/kg T4: 10 g/kg	Improved meat quality and growth performance. Increased the total body weight, no prominent change in FCR	[84]

*Arthrospira platensis*	Japanese quail	480/4 replicates	5	0.5 g/L 1 g/L 2 g/L	Enhanced meat quality and increased the weight in liver, heart, small intestine length, sex organs At 2 g/L, it shows best performance	[58]

*Spirulina platensis*	Japanese quail (*Coturnix coturnix japonica*)	100/4 replicates	8	0.5% 1% 2%	Increased the quality of meat and quality of egg yolk, rate of egg production, and FCR	[85]

*Dunaliella salina* *+* *Arthrospira platensis*	Quail chicks	240/6 replicates	5	DsAp0.5: 0.25 kg/g each; DsAp1: 0.5 kg/g each; DsAp2: 1 g/kg each	Improved the quality of meat, promoted growth performance, and increased the total body weight At DsAp1, best performance for weight of live body and body weight gain	[35]

*Spirulina platensis*	Japanese quail chicks	180/3 replicates	5	2.5 g/kg 5 g/kg 10 g/kg 20 g/kg	Increased body weight and improved meat quality and carcass yield Overall 5 g/kg shows best performance	[59]

In most studies, an intermediate level of inclusion resulted in the most consistent effect. Excessive levels of inclusion were associated with either no improvements or with a reduction in feed efficiency. For example, when fed 2.5% *S. platensis*, the total body weight increased while 7.5%. *S. platensis* caused an increase in feed intake and FCR, indicating that excessive amounts of the feed supplement did not improve feed utilization. The same pattern was found in *A. platensis* in which its supplementation at 4% gave the best results regarding growth performance, carcass composition, and meat quality, while supplementation at 8% adversely affected the same parameters. This suggests that microalgae may be effective in increasing productivity, but their effectiveness may be reduced if the supplementation rate is too high for the birds’ metabolic or digestive system.

## 8. Effects on Egg Quality

A great deal has been studied in the Japanese quail on the use of microalgae as a functional feed to improve egg quality parameters, especially yolk pigmentation and shell quality. The responses on average are more evident for external quality traits than for internal egg components. One definite and well‐established effect is the enhancement of yolk color. In Japanese quails, supplementation with *Schizochytrium* sp. in the diet has been reported to significantly increase the yolk pigmentation and show both linear and quadratic responses over the range of supplementation levels. This effect did not vary with the sampling period which suggests that microalgae‐derived pigments and bioactive compounds, especially carotenoids and polyunsaturated fatty acids, have a direct impact on the enhancement of the yolk color [86, 87]. Likewise, *S. platensis* extract has been shown to enhance the color of eggs even in stressful conditions, indicating its ability to promote pigment deposition and enhance egg appearance quality [88].

The Japanese quails have mixed eggshell quality responses with a moderate to positive short‐term response. In some cases, eggshell thickness has been reported to be temporarily improved after the inclusion of *Schizochytrium*, especially at moderate inclusion rates (0.4%–0.8%), but this effect may be reduced when it is fed for a long period [86]. Other studies, however, show more lasting effects, with some microalgae‐based supplements resulting in substantial increases in eggshell thickness after a few weeks of supplementation [83]. Similarly, quail fed with *Spirulina* showed increased eggshell thickness and shell percentage, which indicates positive mineral utilization and eggshell formation efficiency [88, 89]. But most internal egg quality parameters in Japanese quails do not seem to be influenced by the use of microalgae. In most of the traits, such as albumen percentage, yolk percentage, Haugh unit, yolk index, eggshell strength, egg weight distribution, and component ratios, the differences between the treated and the control groups were not significant [83, 86, 89]. This suggests that microalgae influence visual and structural egg characteristics but show relatively little effect on the overall egg composition. Thus, supplementation with microalgae can be especially effective for improving yolk pigmentation in quails and can possibly increase eggshell thickness and other eggshell quality traits across the board, especially during the early to mid‐feeding trials. However, these characteristics of the internal eggs, more related to the deposition of pigment and shell mineral dynamics, do not significantly change.

## 9. Sustainable Integration of Microalgae Into Quail’s Nutrition

The use of microalgal extract as feed and supplements for quails is not only highly sustainable but also added value to nutrition. Microalgae can be grown in a small area with very little water, even in places unsuitable for normal agricultural production [90]. Microalgae grow vigorously under diverse environmental conditions; however, significant costs can be associated with the establishment and operation of infrastructures for their cultivation in open ponds as well as photobioreactors. A better option would be to use photobioreactors, which allow good control over growth parameters but require a high initial investment not only for purchasing them but also as maintenance costs [91].

Microalgae are renewable energy sources because they reproduce rapidly [92]. Hence, reducing pressure on freshwater resource as sustainable alternative cropping system replaces conventional one. Microalgae are involved in carbon sequestration since CO_2_ is incorporated into its biomass during photosynthetic process, thus reducing GHG emission. Thus, these properties of microalgae enable it to help in pollution control and bioremediation. This makes them an environmental cleanup tool as well as a component of sustainable feed. The cost of producing microalgae is higher than that of other feedstocks, and therefore, processing methods and cultivation systems should be improved to make it commercially feasible. According to one report published in 2018, five taxa yield approximately five kilotons per year with production expenses averaging about 25 k/ton [93].

An additional report placed the annual underutilized global land aggregate microalgae biomass at about 1.985 × 1011 tons [94]. Since they are so small (3–60 μm), harvesting presents another set of problems which requires much energy‐intensive means such as centrifugation [95]. Microalgae have gained substantial commercial importance across various sectors, including nutraceuticals, animal nutrition, and the pharmaceutical industry. The global market value of microalgae is projected to reach approximately 1.143 billion US dollars by 2024. The economic sustainability of using microalgae in poultry feed is less cultivation cost and processing cost per unit of bioavailable nutrient [96].

## 10. Economic and Sustainability Aspects

**FIGURE 5:**
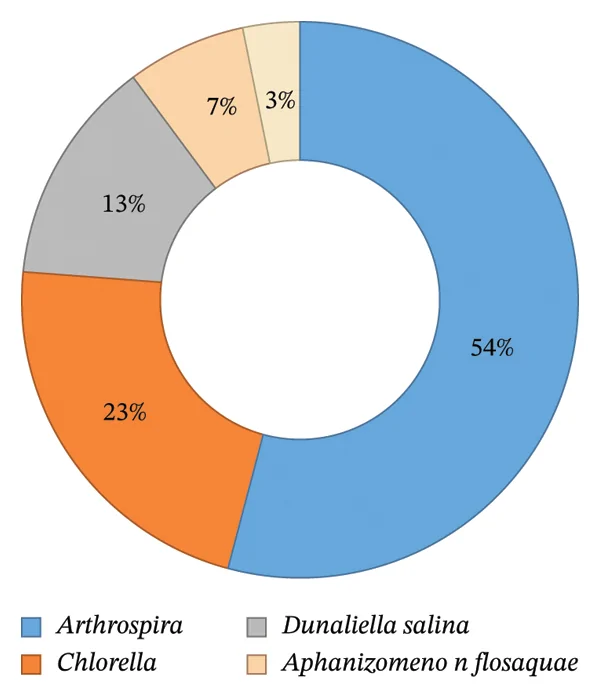
Distribution of commonly used microalgae as feed or its supplements.

Quail feed generally accounts for 70%–80% of the total production cost, hence the major proportion of quail production expenditure. Additional costs are imposed by microalgae: strain selection, cultivation system, whether open ponds or photobioreactors, and downstream processing steps such as harvesting and drying [97]. The nutritional value and production cost must both be evaluated when considering microalgae as an alternative feedstuff [26]. Annual utilization of different microalgal species worldwide indicates the highest use of *Arthrospira* (Figure 5).

Beneficial effects of using microalgae as feed depends on the inclusion level, biomass price, performance improvements, and market premium for functional meat or eggs. For instance, at moderate inclusion rates, economic efficiency increased slightly from 100 to ∼104%, while at higher inclusion levels, efficiency declined due to higher feed costs. Thus, the economic viability of microalgae depends on whether the incremental revenue from improved productivity and product quality outweighs the additional feed cost [27]. The setting up and managing cultivation infrastructures can incur significant expense. However, the use of low‐cost nutrient sources (e.g., wastewater and CO_2_ from industry) and open‐pond systems may reduce production costs. Photobioreactors are more efficient and controlled but require higher initial investment. Therefore, smaller operations, such as quail farms, may find low‐tech or regionally sourced microalgae more feasible [53]. Microalgae supplementation can enrich poultry products with omega‐3 fatty acids, carotenoids, and antioxidants, which adds nutritional value and may justify a market premium. The supplementation of microalgae enriches meat and eggs with beneficial compounds, enhancing their nutritional value. If consumer markets are receptive to such value‐added products (e.g., omega‐3–enriched quail meat), producers could offset higher feed costs through premium pricing [53].

Microalgae, due to their rich nutritional profile and multifunctional biological activity, are emerging as promising sustainable and cost‐effective feed supplements in Japanese quail farming [54]. It can be incorporated into quail feed and have the potential to enhance the production process and improve growth performance, feed conversion ratio, physiological status, carcass quality, and bird health. This makes it a potentially beneficial trait for producers to reduce losses due to poor performance, susceptibility to disease, and environmental stress [53]. The productivity outcomes of the supplementation of diets with microalgae are highly dependent on the inclusion rate, and it has been demonstrated in specific studies for Japanese quail that moderate levels have been associated with the most favorable biological and economic results [25, 50]. For example, low dietary levels of *D. salina* and *A. platensis* supplementation can be used to enhance growth performance, carcass characteristics, and lipid metabolism in quail without significantly increasing production costs [35]. In addition to the production applications, microalgae have valuable sustainability features. Furthermore, microalgae can be grown in a control system throughout the year, which can offer a fairly consistent source of nutrient‐rich biomass [53]. Nevertheless, some factors should still be taken into consideration such as biology of the studied microalgae, methods of processing, level of inclusion in quail diet, availability of microalgae in the region, and level of production before using microalgae in the routine feed of Japanese quail. Thus, it is important to take into account the biological efficacy and practicality when considering the use of microalgae as feed supplements in large‐scale commercial quail production in Japan [53, 98].

## 11. Safety Considerations and Future Perspective

While the use of microalgae as feed for Japanese quail nutrition has demonstrated great potential, it necessitates careful species selection, standardized production processes, and robust quality control measures to guarantee both safety and uniformity [50, 99]. A key factor in the use of microalgae in quail feed is quality assurance due to the variations in nutritional value of microalgae depending on cultivation, processing conditions, and environmental factors. Previous studies have shown that microalgae may bioaccumulate heavy metals and other environmental pollutants under the wrong conditions and some cyanobacteria species may produce unwanted compounds, microcystins, which can impact bird health [54, 100]. Therefore, it is important to carefully screen the microalgal products for their chemical composition, microbial contamination, and toxic residues before incorporating them into the diets of Japanese quail [53, 99]. Additionally, the stability, bioavailability, and safety of the nutrients could be influenced during processing, such as during drying, storage, and extraction, highlighting the importance of standardized manufacturing processes [50]. The second important factor is the level of inclusion, both too high and too low, and it may not be nutritionally balanced and can result in poor bird health and performance. Therefore, further investigations are needed to establish the correct inclusion rates especially for Japanese quail [36, 38]. Further research is also needed to conduct long‐term feeding trials in Japanese quail, evaluate safety of the residues in quail products, develop regulatory guidelines, and develop cost‐effective processing technologies that preserve the nutrient functionality without compromising safety [16, 51]. More studies and greater knowledge of the response of various species, coupled with improved quality control systems, could prove microalgae a valuable functional feed ingredient in commercial quail feeds [50, 53, 55].

Some microalgae types may be filled with unwanted material such as bioactive toxins, purine compounds, and heavy metals which will need thorough risk assessment before they become available as feed supplements [101]. Over 200,000 algal species have been taxonomically described so far, and only around 35,000 of them are reported to be safe to be used as human or animal feed [102]. Some species of microalgae have an extreme ability to bioaccumulate heavy metal element to levels that exceed those of their immediate environments, and others produce neurotoxic or cytotoxic metabolites. The above properties suggest that they may be harmful to the health of the poultry and, more indirectly, to the health of human consumers, which means that serious safety assessments are needed before their inclusion into the feed formulations [103]. Toxicological analysis is thus critical in identifying possible risks that are related to the use of microalgal biomass. Extensive toxicity research has played a pivotal role in the safe and viable use of microalgae as new feed products, and recent studies reflect an interest in the topic of algal toxicity and safety issues [104].

Some of the taxa include toxins like microcystin that can negatively impact the health of poultry and pose a risk to the food safety of humans who consume poultry products [105]. The next emerging issue relates to the possibility of allergenic reactions caused by the microalgal constituents. Though this area of research is still relatively unexplored as a result of the recent introduction of microalgae into animal feeds, allergenicity testing is becoming an essential element in terms of safety [106]. Furthermore, nonprotein nitrogen in the biomass of microalgae can decrease the useful protein value of the biomass to the monogastric animals that do not have ruminal microorganisms able to decompose such sources of nitrogen. This shortcoming underscores the significance of maximizing the amino acid diet and protein digestibility when developing microalgae‐based diets [107].

Although microalgal cell walls are generally indigestible by monogastric animals, they can provide immunomodulatory effects, offering benefits beyond basic nutrition [108]. The initial approaches which have been proposed to improve nutrient bioavailability include enzyme pretreatment to breakdown cell‐wall components and the elaboration of genetically engineered microalgal cultures [109]. Safe use of microalgae in the poultry feed requires close monitoring of toxins and contaminant in addition to a clear labeling to keep consumer consciousness and confidence. There are regulatory systems surrounding microalgae‐based feed which vary according to location. The authorization and safety assessment in the European Union is carried out by the European Food Safety Authority that assesses the efficacy, safety, and environmental impact. In the United States, microalgal feed ingredients are also regulated by the Food and Drug Administration by the Food, Drug, and Cosmetic Act, including novel feed components [110]. Considering these regional differences, the adoption of international unified safety procedures is necessary to protect the health of animals, community safety, and environmental sustainability.

## 12. Conclusion

Microalgae are considered an ecofriendly and multifunctional resource with high potential to revolutionize the feed industry for livestock and poultry. Their nutritional content includes proteins, essential fatty acids, pigments, vitamins, minerals, and other bioactive metabolites that can serve as an alternative to conventional feed ingredients. It has positive effects on growth performance, immune response, antioxidant capacity, gut health, and reproductive system of quails. Moreover, some secondary compounds in microalgae such as sterols, carotenoids, and polysaccharides contribute toward disease resistance and help lower dependency ratio on synthetic antibiotics. Adding microalgae as supplementary feed enriches the nutritional quality parameters as well as enhances the egg and meat production in quails, which in return are beneficial for human health. Globally, microalgae as a sustainable supplementary diet help to promote effective and sustainable food resources, assist more poultry production, and lessen reliance on traditional feed supplies. Microalgae‐based feedstock has developed from a promising idea into a feasible and sustainable alternative for food security and animal nutrition worldwide.

## Funding

No funding was received for this manuscript.

## Conflicts of Interest

The authors declare no conflicts of interest.

## Data Availability

Data sharing is not applicable to this article as no datasets were generated or analyzed during the current study.
